# Caspase-8-mediated PAR-4 cleavage is required for TNFα-induced apoptosis

**DOI:** 10.18632/oncotarget.1634

**Published:** 2014-01-29

**Authors:** Fabian Treude, Ferdinand Kappes, Dirk Fahrenkamp, Gerhard Müller-Newen, Federico Dajas-Bailador, Oliver H. Krämer, Bernhard Lüscher, Jörg Hartkamp

**Affiliations:** ^1^ Institute of Biochemistry and Molecular Biology, Medical School, RWTH Aachen University, Aachen, Germany; ^2^ Faculty of Medicine and Health Sciences, Queens Medical Centre, University of Nottingham, Nottingham, U.K; ^3^ Institute of Toxicology, University Medical Center Mainz, Mainz, Germany

**Keywords:** PAR-4, apoptosis, caspase-8, tumor suppressor, TNFα

## Abstract

The tumor suppressor protein prostate apoptosis response-4 (PAR-4) is silenced in a subset of human cancers and its down-regulation serves as a mechanism for cancer cell survival following chemotherapy. PAR-4 re-expression selectively causes apoptosis in cancer cells but how its pro-apoptotic functions are controlled and executed precisely is currently unknown. We demonstrate here that UV-induced apoptosis results in a rapid caspase-dependent PAR-4 cleavage at EEPD131G, a sequence that was preferentially recognized by caspase-8. To investigate the effect on cell growth for this cleavage event we established stable cell lines that express wild-type-PAR-4 or the caspase cleavage resistant mutant PAR-4 D131G under the control of a doxycycline-inducible promoter. Induction of the wild-type protein but not the mutant interfered with cell proliferation, predominantly through induction of apoptosis. We further demonstrate that TNFα-induced apoptosis leads to caspase-8-dependent PAR-4-cleavage followed by nuclear accumulation of the C-terminal PAR-4 (132-340) fragment, which then induces apoptosis. Taken together, our results indicate that the mechanism by which PAR-4 orchestrates the apoptotic process requires cleavage by caspase-8.

## INTRODUCTION

The tumor suppressor protein prostate apoptosis response-4 (PAR-4) was initially discovered as a pro-apoptotic protein in prostate cancer cells undergoing apoptosis [1]. Mice lacking Par-4 are prone to enhanced tumor development and develop spontaneous tumors as well as displaying an increased susceptibility to hormone- or chemical-induced cancers [2]. Consistent with its role as a tumor suppressor PAR-4 expression is silenced in a well-defined subset of cancers including renal cancers, neuroblastomas, endometrial carcinomas, lung adenocarcinomas, and prostate carcinomas [3-7]. In addition, recent findings by Alvarez and coworkers document that down-regulation of PAR-4 is necessary for tumor cell survival and recurrence of breast cancer following targeted therapy in mouse models and in patients [8]. Down-regulation of Par-4 by oncogenic Ras expression has been shown to require the MEK/ERK MAPK pathway [9] and consistent with this *Par-4* knockout mice cooperate with oncogenic Kras to induce lung adenocarcinomas [6]. Moreover Par-4 was found to be an essential regulator of Hras^G12V^-dependent oncogenic growth in a genome-wide RNAi screen [10].

The protein encoded by the *PAR-4* gene consists of a unique and central SAC (Selective for Apoptosis of Cancer cells) domain encompassing a nuclear localization sequence (NLS) and a C-terminal leucine zipper domain (LZ), which are both 100% conserved in human-, and rodent-orthologs [reviewed in 11]. Interaction with several proteins, including the atypical PKCs (aPKCs), the Wilms' tumor 1 (WT1) protein and DLK/ZIP kinase have been shown to require the leucine zipper domain of PAR-4 [12-14]. On the one hand binding of PAR-4 results in enzymatic inhibition of the aPKC isoforms PKCζ and PKCλ/τ, whereas the interaction with DLK/ZIP kinase and WT1 suggests discrete nuclear functions for PAR-4. The central SAC domain has been identified by serial deletions of PAR-4 and has been described to be indispensable for the pro-apoptotic activities of PAR-4 [15]. It includes a nuclear localization sequence, which promotes nuclear entry and over-expression of this core domain alone induces apoptosis in a variety of cancer cells but does not cause cell death in normal or immortalized cells [15]. Moreover transgenic mice that ubiquitously express the SAC domain of Par-4 are resistant to the development of spontaneous as well as oncogene-induced tumors [16]. These data demonstrate an essential role of the PAR-4 SAC domain for its pro-apoptotic and tumor suppressor activities but how these activities are regulated remains elusive.

Here we show that UV-induced apoptosis leads to a caspase-dependent cleavage of PAR-4 at EEPD131↓G, generating two PAR-4 fragments, the first comprising amino acids 1-131 and the second comprising amino acids 132-340. This cleavage separates the N-terminal part from the C-terminal region that contains the NLS, SAC and the leucine zipper domains. We further demonstrate that TNFα-induced processing of PAR-4 requires caspase-8 and leads to nuclear translocation of the C-terminal part of PAR-4 and thereby induces apoptosis. In summary we have demonstrated that PAR-4 is a novel caspase-8 substrate and provide evidence that PAR-4 cleavage downstream of caspase-8 is required for TNFα induced apoptosis.

## RESULTS

### UV-induced apoptosis results in caspase-dependent PAR-4 cleavage at EEPD131↓G

**Figure 1:**
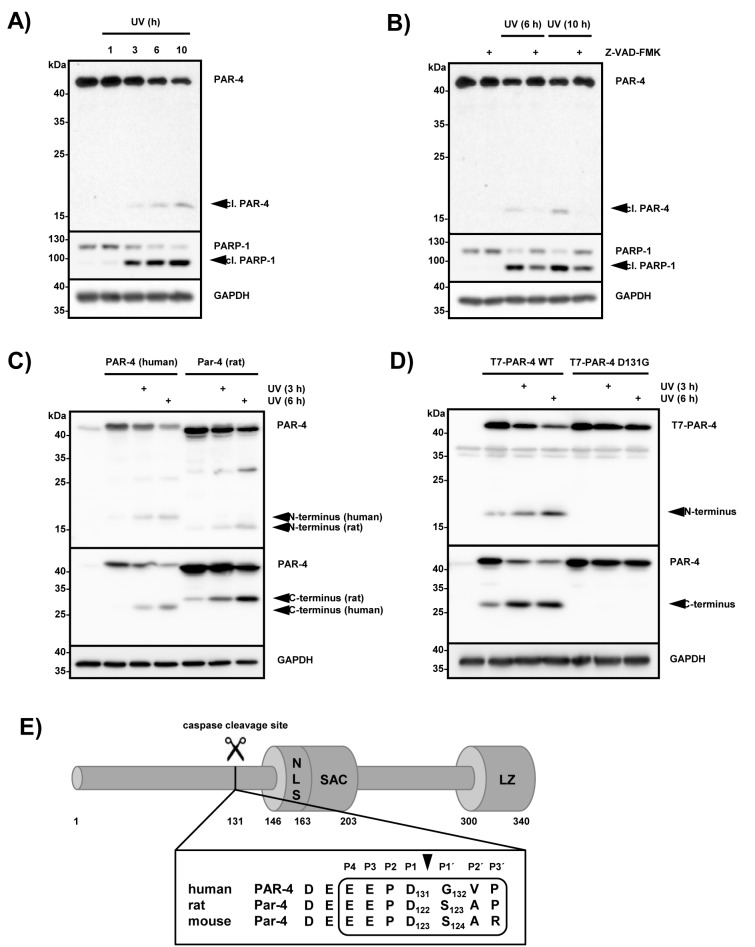
PAR-4 is cleaved during UV-induced apoptosis at position 131 by caspases (A) HeLa cells were treated with UV (20 mJ × cm^−2^ at 254 nm) and incubated for the indicated time points. Cell lysates were analyzed by immunoblotting using antibodies against PAR-4, PARP-1 and GAPDH. A processed form recognized by an antibody that preferentially recognizes the N-terminal part of PAR-4 is indicated (cl. PAR-4). (B) HeLa cells were pre-treated with the pan-caspase inhibitor Z-VAD-FMK (20 µM) for 30 min, then treated as in (A) and incubated for the indicated time points. Lysates were analyzed by immunoblotting as above (A). (C) HeLa cells were transiently transfected with the indicated plasmids (1 µg) and after incubation for 24 h treated with UV light as in (A). After further incubation for the indicated time points, cell lysates were analyzed by immunoblotting using two PAR-4 specific antibodies that preferentially recognize the N-terminal part of the protein (upper panel) or the C-terminal part of PAR-4 (middle panel). (D) HeLa cells were transiently transfected with plasmids encoding T7-tagged PAR-4 or T7-tagged PAR-4 D131G (1 µg each). 24 h after transfection and treatment with UV for the indicated times, whole cell lysates were prepared and analyzed by immunoblotting with T7-specific antibodies recognizing the N-terminus of PAR-4 (upper panel) or PAR-4 specific antibodies that recognize the C-terminal part of the protein (middle panel). (E) Schematic overview of human PAR-4 protein: the caspase cleavage site at position 131 adjacent to the SAC domain (Selective for Apoptosis in Cancer cells, amino acids 146-203); NLS sequence (nuclear localisation signal, amino acids 147-163); LZ motif (leucine zipper, amino acids 300-340). The PAR-4 caspase cleavage site is conserved in human and rodent orthologs with the exception of a Ser instead of Gly at position P1' in mouse and rat Par-4.

Previous findings indicated that PAR-4 selectively induces apoptosis in cancer cell lines including HeLa cells [11]. To further evaluate these findings we treated HeLa cells with UV and analyzed the lysates after the indicated time points using PARP-1 cleavage as a marker for caspase activity (Fig 1A). Within 3 hours of UV treatment efficient PARP-1 cleavage was detectable and at the same time a PAR-4 fragment of ~17 kDa became visible using a PAR-4 amino-terminal antibody, suggesting that this protein may be cleaved during apoptosis (Fig 1A). To investigate whether PAR-4 is hydrolyzed by caspases, HeLa cells were treated with UV in the presence or absence of Z-VAD-FMK, a potent and pan-specific caspase inhibitor [22]. The pre-incubation with Z-VAD-FMK prevented PAR-4 and PARP-1 cleavage in HeLa cells, indicating that UV-induced PAR-4 hydrolysis is caspase-dependent (Fig 1B). To analyze if UV-mediated PAR-4 processing was species specific we overexpressed human and rat PAR-4 in Hela cells and treated the cells with UV. Figure 1C shows that UV treatment resulted in the generation of a ~17 kDa N-terminal and a ~28 kDa C-terminal fragment for human PAR-4 and a ~15 kDa N-terminal and a ~30 kDa C-terminal fragment for rat Par-4, indicating the existence of a single cleavage site in both species. We scanned the PAR-4 sequence for potential caspase cleavage sites on the CASVM server (Server for SVM prediction of caspase substrate cleavage sites; www.casbase.org), which revealed a potential cleavage site at EEPD131↓G in the human protein [23]. To validate this finding we mutated Asp131 to Gly, overexpressed PAR-4 and PAR-4 D131G in HeLa cells and incubated them for the indicated times after UV treatment. Figure 1D demonstrates that a PAR-4 D131G mutant was resistant to UV-induced processing and no cleavage products were generated. These data confirmed the existence of a single caspase cleavage site at residue EEPD131↓G in human PAR-4. The cleavage site separates the N-terminal region from the SAC and leucine zipper domains (Fig 1E). This sequence is conserved in rat and murine Par-4, albeit slightly shifted towards the N-terminus, explaining at least in part the altered mobility of cleaved rat Par-4 (Fig 1C and 1E).

### Inducible expression of PAR-4 but not PAR-4 D131G interferes with cell proliferation

**Figure 2:**
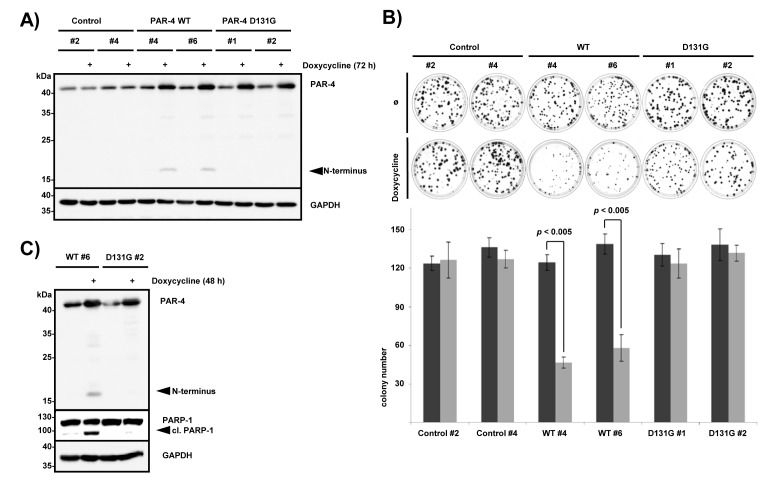
PAR-4 cleavage leads to inhibition of cell growth (A) Individual established clones of HeLa Flp-In PAR-4 wild-type (WT), PAR-4 D131G and control (empty vector) T-REx cells were treated with 100 ng/ml doxycycline for 72 h. Protein expression was analyzed by immunoblotting using the indicated antibodies. N-terminal PAR-4 fragments are indicated. (B) For the colony formation assay, 200 cells were seeded and grown for 12 days ± doxycycline prior to staining with methylene blue. Two different clones each for PAR-4 WT, PAR-4 D131G and control cells were analyzed (upper panel). Average values from three independent experiments are displayed. Doxycycline treated cells are shown in light grey. Error bars indicate ± SD. P-value was obtained by two-tailed Student's t-Test, comparing doxycycline treated with non-treated cells (lower panel). (C) Hela Flp-In PAR-4 wild-type and PAR-4 D131G cells were grown for 2 days ± doxycycline. Protein lysates were analyzed with the indicated antibodies.

We were next interested if the observed PAR-4 cleavage exhibits any biological effects. Therefore, we generated multiple HeLa Flp-In T-REx cell clones, which either express PAR-4 wild-type or PAR-4 D131G from the identical locus after the addition of doxycycline (Fig 2A). Subsequent analysis of the growth characteristics of stable cell clones in colony formation assays revealed a marked reduction of colony number and also colony size upon induced expression of wild-type PAR-4 but not PAR-4 D131G (Fig 2B). This was observed with four individual clones, and shown here for two clones that express either empty vector (#2 and #4), PAR-4 wild-type (#4 and #6) or PAR-4 D131G (#1 and #2). Although the inducible expression of PAR-4 wild-type and PAR-4 D131G was comparable (Fig 2A), we noted that expression of PAR-4 wild-type led to the generation of a caspase cleavage fragment (Fig 2A). This suggested that moderate overexpression of PAR-4 was sufficient to induce caspase activation as observed previously [15]. Therefore we compared the capacity of PAR-4 wild-type and PAR-4 D131G to induce apoptosis. Figure 2C illustrates that only expression of PAR-4 wild-type but not the caspase cleavage resistant mutant led to increased PARP-1 cleavage indicating that caspase processing of PAR-4 is necessary to activate its pro-apoptotic properties.

### PAR-4 is a substrate of caspase-8

**Figure 3:**
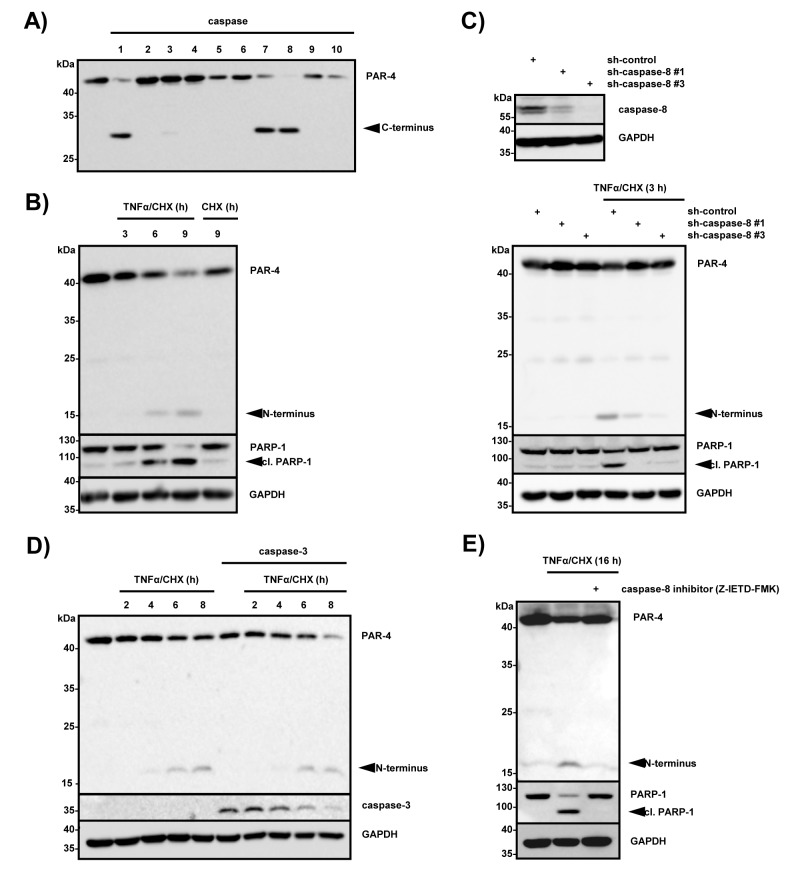
PAR-4 is a substrate of caspase 8 (A) *In vitro* caspase assay using FLAG-tagged PAR-4 wild-type and recombinant human caspases 1-10. The assay was stopped after 30 min by adding sample buffer, and the proteins were subjected to immunoblotting using a PAR-4 antibody recognizing the C-terminal part of the protein. (B) HeLa S3 cells were stimulated with TNFα (10 ng/ml) in combination with CHX (0,5 µg/ml) for the indicated time points and with CHX alone for 9 h. Cell lysates were analyzed by immunoblotting with the indicated antibodies and PAR-4 cleavage is indicated. (C) Cell lysates from HeLa S3 control (sh-control) or caspase-8-deficient (sh-caspase-8 #1 and #3) were analyzed by immunoblotting with caspase-8 and GAPDH antibodies (upper panel). HeLa S3 sh-control or sh-caspase-8 #1 and #3 cells were incubated with TNFα (10 ng/ml) in combination with CHX (0,5 µg/ml) for 3 h. Protein lysates were analyzed by protein immunoblotting with antibodies against PAR-4, PARP-1 and GAPDH (lower panel). (D) Caspase-3-deficient MCF-7 cells and MCF-7 cells stably expressing caspase-3 were stimulated with TNFα (10 ng/ml) and CHX (0,5 µg/ml) for the indicated time points and protein lysates were analyzed by immunoblotting with PAR-4, caspase-3 and GAPDH-specific antibodies. PAR-4 cleavage products are indicated. (E) MCF-7 cells were pre-incubated with 50 µM caspase-8 inhibitor (Z-IETD-FMK) for 30 min and subsequently stimulated with TNFα (10 ng/ml) and CHX (0,5 µg/ml) for 16 h. Cell lysates were analyzed with the indicated antibodies via immunoblotting and cleavage of PAR-4 is indicated.

In order to identify caspases that are capable of cleaving PAR-4, immunoprecipitated Flag-tagged PAR-4 was subjected to a caspase cleavage assay with recombinant caspases 1 to 10 (Fig 3A). Caspase-1, -7 and -8 were able to cleave PAR-4 to various degrees *in vitro*, with caspase-8 being the most efficient to hydrolyze full length PAR-4 (Fig 3A). The tumor necrosis factor (TNFα) receptor family is an established mediator of the extrinsic apoptotic pathway and stimulates apoptosis through death-inducing signaling complex (DISC) formation, which includes engagement and activation of caspase-8 [24]. To study the role of caspase-8 in PAR-4 processing we stimulated HeLa S3 cells for various times with TNFα and cycloheximide and found that TNFα-induced signaling led to simultaneous PAR-4 and PARP-1 cleavage (Fig 3B). Next, we sought to investigate if caspase-8 is required for TNFα/CHX induced PAR-4 cleavage. For this purpose, we created HeLa S3 cell lines using lentiviral delivery of shRNA constructs either expressing two caspase-8-specific shRNAs (sh-caspase-8 #1, #3) or a non-silencing shRNA, which serves as a control (sh-control). The expression of caspase-8 was strongly reduced in HeLa S3 cells transduced with caspase-8 shRNA #1 and #3 as shown in Figure 3C (Fig 3C, upper panel). Stimulation with TNFα/CHX only induced PAR-4 and PARP-1 cleavage in the presence of caspase-8 indicating that PAR-4 is downstream of caspase-8 (Fig 3C, lower panel). Together these findings suggest that PAR-4 is a direct target of caspase-8. Recently Chaudhry and coworkers showed that PAR-4 is a substrate of caspase-3 and demonstrated that PAR-4 cleavage does not occur after cisplatin treatment of caspase-3-deficient MCF-7 cells [25]. As our *in vitro* experiment showed only very weak activity of caspase-3 towards PAR-4 (Fig 3A), we addressed the role of caspase-3 in our cells. Therefore we measured TNFα-induced PAR-4 cleavage in caspase-3-deficient MCF-7 cells and in caspase-3 reconstituted cells (Fig 3D). Stimulation of MCF-7 cells with TNFα led to PAR-4 cleavage regardless whether caspase-3 was absent or present indicating that TNFα-induced PAR-4 processing is caspase-3 independent (Fig 3D). Moreover pre-treatment of MCF-7 cells with the caspase-8 specific inhibitor Z-IETD-FMK demonstrated that TNFα-induced PAR-4 cleavage was caspase-8 dependent (Fig 3E).

### Caspase-8-mediated cleavage of PAR-4 leads to apoptosis and to nuclear accumulation of the C-terminal fragment of PAR-4

**Figure 4:**
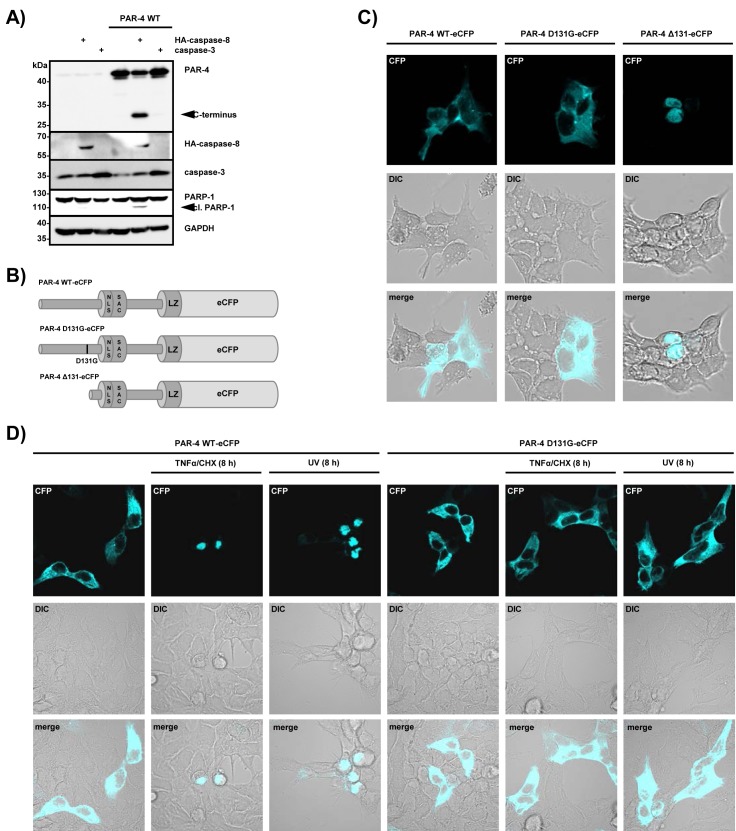
Caspase-8-mediated PAR-4 cleavage promotes apoptosis through nuclear translocation (A) HEK 293 cells were transiently transfected with plasmids driving expression of PAR-4 wild-type (1 µg), caspase-8 or caspase-3 (50 ng each) for 16 h. Protein lysates were analyzed by protein immunoblotting with the indicated antibodies. PAR-4 cleavage was analyzed with antibodies recognizing the C-terminal part of the protein. (B) Schematic representation of PAR-4 wild-type, PAR-4 D131G and PAR-4 Δ131 fused to eCFP as used for (C). (C) HEK 293 cells were transiently transfected with the plasmids indicated above (B) and after 16 h analyzed by confocal microscopy. DIC: differential interference contrast. (D) HEK 293 cells were transiently transfected as above and after 16 h incubation stimulated with UV (20 mJ × cm^−2^ at 254 nm) or TNFα (10 ng/ml) and CHX (0,5 µg/ml). 8 h after stimulation cells were analyzed by confocal microscopy.

To further investigate functional consequences of caspase-8-mediated PAR-4 processing, we co-expressed wild-type PAR-4 and caspase-8 in HEK 293 cells. Forced expression of caspase-8 and PAR-4 on there own has been shown to trigger apoptosis and therefore we carefully titrated the amounts to generate conditions under which overexpression of each does not result in the induction of apoptosis. Figure 4A demonstrates that expression of caspase-8 and PAR-4 on its own does not induce apoptosis but co-expression of the two proteins induced PAR-4 and PARP-1 cleavage, indicating induction of apoptotic cell death. In contrast, co-expression of caspase-3 and PAR-4 did not result in PAR-4 cleavage or induction of cell death (Fig 4A), again underscoring the functional relation between caspase-8 and PAR-4. Induction of apoptosis in cancer cell lines by expression of the central SAC domain of PAR-4 has been shown to require nuclear localization (Fig 1E) [15]. To study the localization of the PAR-4 cleavage product containing the SAC and leucine zipper domains, we generated PAR-4 mutants with a C-terminal eCFP tag (Fig 4B). Whereas PAR-4 wild-type and PAR-4 D131G localized to the cytosol as expected, the PAR-4 mutant lacking the amino-terminal part localized to the nucleus (Fig 4C). Moreover, stimulation with TNFα/CHX or UV resulted in nuclear accumulation of PAR-4 wild-type, but was prevented in cells expressing PAR-4 D131G (Fig 4D). These data indicate that caspase-8-mediated processing of PAR-4 might result in the nuclear accumulation of the C-terminal fragment of PAR-4 and induction of cell death.

### TNF-induced apoptosis requires caspase-8-mediated processing of PAR-4

**Figure 5:**
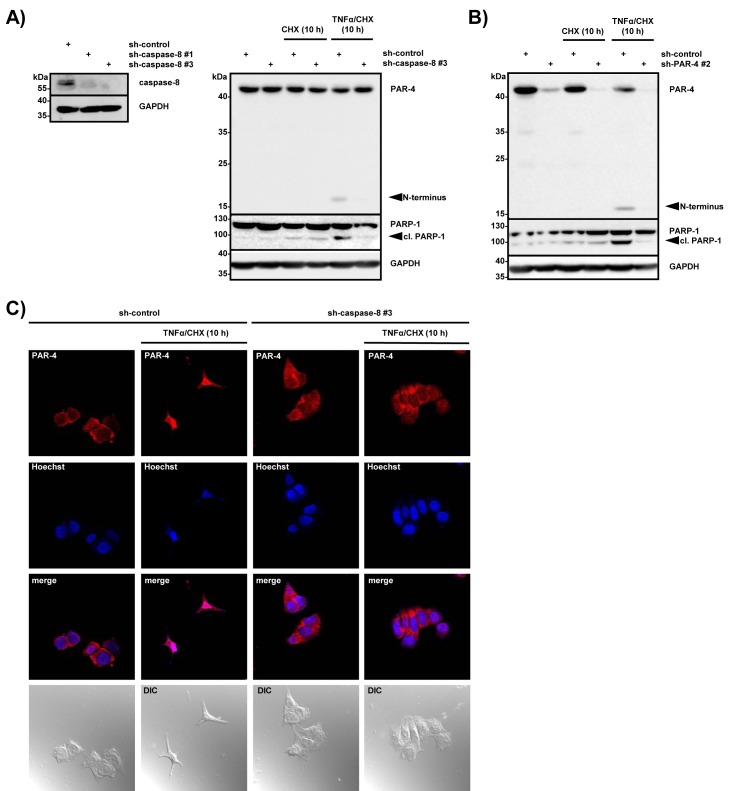
PAR-4 is required for TNFα-induced apoptosis (A) Cell lysates from MCF-7 control (sh-control) or caspase-8 deficient (sh-caspase-8 #1 or #3) cells were analyzed by immunoblotting using antibodies against caspase-8 and GAPDH (left panel). MCF-7 control and caspase-8 deficient cells were incubated with CHX (0,5 µg/ml) or TNFα (10 ng/ml) in combination with CHX (0,5 µg/ml) for 10 h. Cell lysates were analyzed by protein immunoblotting with antibodies against PAR-4, PARP-1 and GAPDH and (right panel). N-terminal PAR-4 cleavage products are indicated. (B) MCF-7 control (sh-control) or PAR-4 deficient (sh-PAR-4 #2) cells were treated with CHX (0,5 µg/ml) alone or TNFα (10 ng/ml) in combination with CHX (0,5 µg/ml). After 10 h of incubation cell lysates were analyzed by immunoblotting with antibodies against PAR-4, PARP-1 and GAPDH. N-terminal PAR-4 cleavage products are illustrated. (C) MCF-7 control (sh-control) and caspase-8 deficient cells (sh-caspase-8 #3) were stimulated for 10 h with TNFα (10 ng/ml) and CHX (0,5 µg/ml), immunostained with a C-terminal PAR-4 antibody (red), treated with Hoechst (blue) for nuclear staining and analyzed by confocal microscopy. DIC: differential interference contrast.

Next we wanted to analyze whether caspase-8-mediated PAR-4 cleavage is required to trigger TNFα-induced cell death in caspase-3-deficient MCF-7 cells. Therefore we generated caspase-8-deficient MCF-7 cell lines and control cell lines using lentiviral delivery as described above (for knockdown efficiency see Fig 5A, left panel). The cells were then treated with CHX and TNFα/CHX and induction of apoptosis was measured by PARP-1 cleavage. While sh-control cells underwent apoptosis and showed PAR-4 processing after TNFα/CHX stimulation, caspase-8-deficient cells failed to do so (Fig 5A, right panel). CHX treatment alone was not sufficient to induce PAR-4 processing and apoptosis. To investigate if PAR-4 expression was required for the induction of apoptosis in response to TNFα/CHX, we compared PAR-4-deficient with control MCF-7 cells stimulated with TNFα/CHX. Apoptosis was induced in sh-control cells but was significantly inhibited in PAR-4-depleted cells (Fig 5B) and similar results were also obtained in HeLa S3 cells (data not shown). To expand on these findings we analyzed the localization of endogenous PAR-4 after TNFα/CHX induced apoptosis with a C-terminal PAR-4 antibody. Under apoptotic conditions PAR-4 localized to the nucleus while this effect was largely inhibited in caspase-8-knockdown cells (Fig 5C). These results suggest that PAR-4 cleavage is a direct consequence of caspase-8 activation and is required for nuclear accumulation and induction of apoptosis mediated by the C-terminal fragment of PAR-4.

## DISCUSSION

PAR-4 is a multi-domain protein and functions as a tumor suppressor in a subset of human cancers. It contains pro-apoptotic activities but the signaling pathways functioning upstream of PAR-4 are ill defined. In this study, we found that PAR-4 is cleaved upon UV- and TNFα-induced induction of apoptosis at EEPD131↓G and this cleavage site was preferentially recognized by caspase-8. Furthermore, caspase-8-mediated PAR-4 cleavage is critical in regulating cell death triggered by TNFα, which indicates that PAR-4 functions downstream of caspase-8.

Like many proteases caspases display cleavage-site specificity and share some extent of amino acid specificity adjacent to the site of hydrolysis, providing some degree of substrate site selectivity. Caspases have a strict requirement for an aspartate in the P_1_ position, with P_1_-P_1_' being the cleavable bond (P_4_-P_3_-P_2_-P_1_-↓-P_1'_). Differences in the amino acids in the P_4_, P_3_, P_2_ positions mainly determine caspase specificity [26]. The optimal caspase-8 cleavage site was determined to require P_4_ (L, V, D, E) P_3_ (E) P_2_ (I, T, V) P_1_ (D) P_1_' (G, S) [27,28]. The PAR-4 cleavage site EEPD131↓G fulfills these requirements except for position P_2_. Human and rodent PAR-4 cleavage site motifs are conserved except for position P_1_' (Fig 1E). The P_1_' residue requires small and uncharged amino acids (Gly, Ser, Ala) and instead of a Gly at P_1_' in human PAR-4, mouse and rat Par-4 contain a Ser, which fits with the amino acids required for a bona fide caspase-8 substrate.

The Cys protease caspase-8 initiates apoptotic cell death in response to cell surface activation of TNF death receptors by undergoing autocleavage and then initiating processing of executioner caspases-3 and -7 [29]. Although large-scale proteomics in cells have shown that caspases cut hundreds of proteins generally at a single site, only a few proteins, such as Bid, p28 Bap31, RIP-1, osteopontin and CYLD are reported as caspase-8 substrates [30-34]. The tumor suppressor protein PAR-4 predominantly comprises an intrinsically disordered protein, with ordered segments in the C-terminal domains of the protein [35]. In this study we demonstrate that caspase-8 cleaves PAR-4 after Asp 131, thereby separating the unstructured N-terminus from the C-terminal part, which includes the NLS-containing SAC domain and the leucine zipper. Therefore, the C-terminal fragment possesses all the domains required for nuclear translocation and induction of apoptosis (Fig 1E). Previous studies have shown that nuclear entry of the SAC domain is essential for PAR-4-induced apoptosis [15]. Our own immunofluorescence data demonstrate that cleavage of PAR-4 markedly enhances nuclear targeting of the C-terminal cleavage product but how this shuttling process is regulated is still unknown. One possible mechanism is provided by 14-3-3 proteins, which are phospho-serine/phospho-threonine binding proteins. PAR-4 has been shown to associate with the mainly cytosolic 14-3-3 sigma isoform [36,37], which also interacts and sequesters the transcription factor YAP in the cytosol and thereby prevents it from activating p73-induced apoptosis in the nucleus [38]. It can therefore be speculated that caspase-8-activated hydrolysis of PAR-4 interferes with 14-3-3-mediated cytoplasmic retention of PAR-4, thereby inducing nuclear targeting of the C-terminal cleavage product.

In a recent study by Chauhdry et al., the authors identified PAR-4 to be a substrate of caspase-3 during apoptosis and demonstrated that cisplatin-induced PAR-4 cleavage is abrogated in caspase-3-deficient MCF-7 cells [25]. We analyzed the ability of caspase-1 to -10 to hydrolyze PAR-4 *in vitro.* Only caspase-1, -7 and -8 were able to efficiently cleave PAR-4, while caspase-3 showed only very weak activity. Moreover, only co-expression of PAR-4 with caspase-8, but not with caspase-3, led to PAR-4 cleavage and induction of apoptosis in HEK 293 cells. To verify these data we also utilized caspase-3-deficient MCF-7 breast cancer cells and analyzed PAR-4 cleavage after stimulation of TNF death receptors. The inflammatory response of cells to the pleiotropic cytokine TNFα can be switched to apoptosis by the addition of protein synthesis inhibitors that shut down the synthesis of the endogenous caspase-8 inhibitor c-FLIP leading to caspase-8 activation [39]. Our combined results demonstrate that TNFα/CHX-induced PAR-4 cleavage in MCF-7 cells requires caspase-8, but is caspase-3 independent. Together, our data support a critical role for caspase-8 in TNFα-induced hydrolysis of PAR-4.

As PAR-4 functions as a tumor suppressor in a subset of human cancers [11] and can be cleaved by caspase-8, our findings might aid in explaining some of the controversial functions of caspase-8 in tumorigenesis [40]. Caspase-8 has been reported to be silenced in a subset of human cancers owing to gene deletion, mutation or promoter hypermethylation, all resulting in a reduced capacity to trigger apoptosis [reviewed in 40]. This strongly suggests that caspase-8 possesses tumor suppressor functions and indeed caspase-8 deficiency facilitates cellular transformation [41]. Thus, we speculate that a role of caspase-8 deficiency in tumorigenesis may be in part due to its failure to cleave and induce PAR-4 translocation and activation.

In summary, our data demonstrate that PAR-4 is a novel substrate of the initiator caspase-8 and is cleaved during TNFα- and UV-induced apoptosis. Furthermore, we provide evidence that regulation of PAR-4 through its hydrolysis by caspase-8 during TNFα-induced apoptosis is an essential step for the induction of cell death in some cancer cells. Therefore, our observations provide evidence for a novel mechanism of the regulation of the pro-apoptotic properties of the tumor suppressor protein PAR-4 and future studies will address which pathways are downstream of caspase-8/PAR-4.

## MATERIALS AND METHODS

### Cell culture and transfections

All cell lines were cultured at 37°C with 5% CO_2_, using either Dulbecco's modified Eagle's medium-Glutamax I (HeLa, HeLa S3 and HEK 293 cells) or RPMI 1640 medium (MCF-7 cells) supplemented with 10% fetal calf serum. Caspase-3 reconstituted MCF-7 cells were cultured as described before [17]. For the selection of stable HeLa Flp-In T-REx cells 5 µg/ml blastidicidine and 100 µg/ml hygromycin were added to the medium. To achieve stable PAR-4 and caspase-8 knockdowns the following plasmids from the Thermo Scientific GIPZ lentiviral shRNA library were used: caspase-8 (V2LHS_112730 and V2LHS_112733), PAR-4 (V2LHS_152662) and non-silencing control (RHS4346). Lentiviral transduction procedures were carried out as described before [18] and 1 µg/ml puromycin was added to the medium of lentiviral transduced HeLa S3 and 2 µg/ml puromycin to the medium of MCF-7 cells. Transient transfection was achieved by calcium phosphate precipitation (HEK 293 cells) [19], or by using Lipofectamine® 2000 (Invitrogen) according to the manufacturer's instructions. Prior to UV (20 mJ × cm^−2^ at 254 nm) treatment cells were washed once with PBS. Cells were treated with 10 ng/ml human TNFα (Peprotech) in combination with 0,5 µg/ml cycloheximide (Sigma). Pretreatment with 20 µM pan-caspase inhibitor Z-VAD-FMK (Calbiochem) or 50 µM caspase-8 inhibitor Z-IETD-FMK (Santa Cruz) was performed 30 min prior further stimulation.

### Stable cell lines

HeLa Flp-In T-Rex cells have been described previously [19] and were transfected with pcDNA5/FRT/TO-PAR-4wt or the respective mutant and pOG44 (Invitrogen). The transfected cells were selected in media containing 5 µg/ml blasticidin and 100 µg/ml hygromycin. Monoclonal cell lines were established after initial selection. Protein expression was induced by treating the cells with 100 ng/ml doxycycline for 72 hours.

### Plasmids and mutagenesis

The expression construct pcDNA3-T7-PAR-4 was generated by amplification of the *PAR-4* cDNA and subcloned into pcDNA3-T7. pcDNA3-T7-PAR-4-D131G was constructed by site-directed mutagenesis with the QuickChange kit (Stratagene) and confirmed by DNA sequencing. PcDNA5/FRT/TO-PAR-4wt or D131G were constructed using pcDNA5/FRT/TO (Invitrogen) and inserts from the respective pcDNA3-T7-PAR-4 vectors. PcDNA5/FRT/TO-PAR-4(132-340) was created using 5′-GTTTAAGCTTATGGGCGTCCCAGAGAAG-3′ sense primer and 5′-TTTGGATCCCTACCTGGTCAGCTGACC -3′ antisense primers. PAR-4(132-340) was then subcloned into the HindIII/AgeI sites of pcDNA5/FRT/TO/ΔN-STAT-CFP vector using sense 5′-TTTAAGCTTCGATGGGCGTCCCAGAGAAGGG-3′ and antisense 5′-GCTCACCGGTATGATCCTGGTCAGCTGACCC AC-3′ primers to generate pcDNA5/FRT/TO-PAR-4(132-340)-eCFP. To create pcDNA5/FRT/TO-PAR-4wt-eCFP and pcDNA5/FRT/TO-PAR-4-D131G-eCFP constructs, PAR-4wt and PAR-4-D131G were subcloned into the pcDNA5/FRT/TO-PAR-4(132-340)-eCFP vector using HindIII/BglII/SnaBI sites. PAR-4wt-Flag was subcloned into the HindIII/BamHI sites of pcDNA5/FRT/TO vector using sense 5′-TTTAAGCTTCGATGGCGACCGGTGGCTAC-3′ and antisense 5′- GAGTGGATCCTCATTTGTCGTCATCGT CTTTGTAGTCCCTGGTCGACTCACCCAC-3′ primers. pcDNA3-Par-4wt (rat) and pcDNA3-caspase-3wt have been described before [20, 17, 21]. pcDNA3-caspase-8wt-HA was constructed by site-directed mutagenesis using pcDNA3-caspase-8-C360A-HA (gift of Guy Salvesen; Addgene plasmid #11818)

### Antibodies

PAR-4 was detected via three different polyclonal antibodies. The C-terminus of PAR-4 was detected by using the anti-PAR-4 antibodies (Cell Signaling, #2328) or anti-PAR-4 (Abcam, #49155), while the N-terminal part was detected using anti-PAR-4 antibodies (Santa Cruz, #1807). Additional antibodies that were used are anti-PARP-1/ARTD-1 (Cell Signaling, #9542), anti-GAPDH 6C5 (Santa Cruz, #32233), anti-HA Y11 (Santa Cruz, #805), anti-caspase-3 (Cell Signaling, #9662) and anti-T7 (Novagen, #69522). Secondary antibodies used were horseradish peroxidase-labeled IgG goat anti-rabbit (#P0448) and rabbit anti-mouse (#P0161) from Dako. For confocal microscopy secondary goat anti-rabbit IgG conjugated with Alexa Fluor® 555 dye (Invitrogen, #A31572) was used.

### Colony formation assay

2×10^2^ cells expressing pcDNA5/FRT/TO-PAR-4wt, pcDNA5/FRT/TO-PAR-4-D131G or the vector control were seeded in 6 cm dishes in duplicates. Protein expression was induced by addition of 100 ng/ml doxycycline with consecutive medium changes every three days. On day 12, the cells were washed once in PBS and subsequently stained with 0.2% methylene blue in methanol for 30 minutes. After washing, dishes were dried and pictured for documentation.

### Caspase assay

Lysates used for immunoprecipitation of proteins subjected to *in vitro* caspase treatment were generated in TLB lysis buffer (50 mM Tris/HCl, pH 7.4, 150 mM NaCl, 1 mM EDTA, 1% Triton-X-100 and a protease inhibitor cocktail). The lysates were incubated with FLAG M2 beads (Sigma) for 3 hours at 4°C, washed and directly used in a caspase assay. 0.1 unit/enzyme recombinant human caspases (PromoKine) were incubated with PAR-4 in 30 µl caspase reaction buffer (50 mM HEPES, pH 7.2, 50 mM NaCl, 0,1% CHAPS, 10 mM EDTA, 2.5% glycerol, 5 mM DTT). After incubation for 30 min at 37°C fragments were analyzed by immunoblotting.

### Indirect immunofluorescence and confocal microscopy

Cells were grown on glass coverslips (18 mm) in 12 well plates, washed with PBS and fixed with 4% paraformaldehyde / PBS for 30 min. Cells were permeabilized with PBS containing 0,1% Triton-X-100 for 30 min and blocked in 3% bovine serum albumin (BSA) in PBS for 1 h. PAR-4 was stained with PAR-4 specific antibodies (Abcam, 1:100) and visualized with secondary Alexa Fluor® 555 conjugated antibodies (1:1000). Hoechst was added and coverslips were mounted with ImmuMount (Thermo Scientific). Images were examined with a Zeiss LSM 710 confocal microscope with a LDC-apochromat 40/1.1 water objective. ZEN 2009 software (Zeiss) was used for image editing.
